# Pediatrics

**Published:** 1905-05

**Authors:** Maud J. Frye

**Affiliations:** Buffalo, N. Y.


### PROGRESS IN MEDICAL SCIENCE.

### Pediatrics.

Conducted by MAUD J. FRYE, M. D., Buffalo, N. Y.

### RECURRENT VOMITING.

Rochford, (*Archives of Pediatrics,* December, 1904,) discusses at length the subject of recurrent vomiting in children. The article is too long to be presented even in abstract, but the following is gleaned from it:

Heredity is the most important predisposing factor. A family history of gout or migraine is present in nearly every case. Nearly all of these patients are constipated. Mental overwork and nerve excitement, especially when combined with indoor life and bad ventilation are important predisposing factors. Nearly all cases occur among the heredity rich and refined. Dr. Rochford believes that both auto- and intestinal toxins play a rôle in producing this symptom group and that it is not always produced by the same toxins. A functional incompetence of the liver is an all-important factor. Of exciting causes overeating is one of the most potent. All reflex irritations may be classed as exciting causes. The urine becomes scanty. It is very concentrated and highly acid. The xanthin bodies are in great excess. Albumin may be present in small quantities, but this is uncommon. Acetone and indican are present in all of the severer cases. Diacetic and oxybutyric acids have been found.

Passing by the description of symptoms it may be noted that intestinal obstruction offers the greatest difficulty in differential diagnosis. Absence of pain and of bloody mucus in the stools and of any tumor help to clear the situation.

The prognosis, both as regards the attack and the prevention of recurrence, is good. Fatal cases have been noted.

Treat the attack by the use of calomel and bicarbonate of soda, followed by a saline laxative, if the stomach be not too irritable. Four or five hours later give benzoate of soda from 3 to 8 grains every two or three hours. No food should be given. Water may be allowed. If the vomiting precludes stomach medication try the calomel and soda and give every eight to twelve hours a high rectal enema of normal salt solution or bicarbonate of soda; a tablespoonful to a pint. In cases of extreme prostration saline solution may be used subcutaneously. It is sometimes necessary in these cases to use morphine, 1-10 to 1-20 grain, hypodermatically.

The curative treatment consists in an outdoor life, with moderate exercise in a suitable climate. As a rule, the children should be taken out of school and lead quiet lives. The diet should be carefully restricted. The following foods may be allowed: milk, cocoa, vegetable soups, well-cooked vegetables, cereals, cooked fruits, eggs, fish, mutton, chicken, and occasionally beef. An excess of any food must be guarded against. The habit of water drinking between meals should be cultivated. Sources of reflex irritation should be sought for and removed. Constipation must be relieved, first by the sulphate and phosphate of soda, later by mixtures of rhubarb and cascara. For the medical treatment the wintergreen salicylate of soda and the benzoate of soda are our best remedies. These may be given for months at a time.

---

### RECTAL EXAMINATION IN CHILDREN.

McKenzie, (*British Journal of Children's Diseases*, December, 1904,) reports a case of appendicitis in a child discovered by this method of examination. The child, aged 4 years, suffered from a high temperature and general malaise. The next day he complained of stomachache and pain on micturition, but from the refractory nature of the child nothing could be made out by abdominal examination. Per rectum the bladder was found to be full and fluctuating. The patient was anesthetised and the child catheterised under the impression that some bladder trouble was responsible for the disorder. A rectal examination with one hand on the abdomen revealed a tumor in the right side of the pelvis, which operation a few hours later proved to be the appendix already perforated. A delay of a few hours more in diagnosis would have cost a life.

Rectal examination in a child permits quite a complete investigation of the condition of the abdomen. To obtain a satisfactory result the patient must be anesthetised.

### GONORRHEAL SALPINGITIS IN A CHILD.

BIDWELL, (*British Journal of Children's Diseases,*) reports a case in a child, aged 6 years. Microscopic examination of the vaginal discharge showed gonococci in considerable numbers. The child was very ill with great pain and restlessness. She refused nourishment. The abdomen was slightly swollen and distended and rectal examination revealed a very tender swelling in the left side of the pelvis. Operation was done and both tubes were found full of pus and were removed. The ovaries were left. Later, a cureting of the uterus was required for cure of endometritis. Following this, the child made a perfect recovery. The case was complicated by acute inflammation of the tendon sheath of the right foot, undoubtedly a form of gonorrheal rheumatism.

Bidwell advises that when a child is seen with a vaginal discharge, which is proved to be gonorrheal, the patient should be anesthetised and, if the discharge is coming from the uterus, cureting should be done to prevent the spread of the disease to the tubes. Salpingectomy should be a last resort, since the functional result is sterility. In his case the ovaries were left that development might not be arrested.

---

### PHYSICAL SIGNS IN CHILDREN.

MCHAMILL AND LEBONTILLIER, (*Jour. Am. Med. Assn.,* January 7, 1905,) report the results of a series of observations, relating to normal or functional conditions, some of which have not been sufficiently emphasised in the literature, and others of which are at variance with the results reported. An area of impaired resonance, they find, exists normally under the left clavicle. This is present until the ninth or tenth and not infrequently till the thirteenth year. It is always present at the inner third of the clavicle and occasionally extends outward to the mid-clavicular line. It extends downward to the first interspace and not infrequently blends into cardiac dulness.

The bronchial type of breathing is ordinarily heard over the root of the lungs, and varies greatly in different children. Posteriorly, it is usually limited to the interscapular space and supraspinous fossæ. It is often conveyed to the entire scapular area and it can sometimes be heard just above and in front of the angle of the scapula, about in the posterior axillary line,—as a high-pitched, distant, bronchial murmur, which is sometimes so misleading as to be mistaken for the bronchial breathing of consolidation. When heard here, it is usually bilateral, assuming that the child is examined in a position which renders breathing equal on the two sides. It is much more common in children under 6 years of age, although noted after this period.

It has been stated that placing a child on a pillow or mattress will to some extent deaden the percussion note. These observers further state that a striking difference between the two sides occurs when the child is examined lying on its side with the dependent surface of the chest in intimate contact with the bedding. Under these circumstances, the elasticity of the chest wall is destroyed, the lung is compressed, air enters it inadequately and, in consequence, there results a striking increase in the height of the pitch of the percussion note, which, when compared with the note on the opposite side, proves very misleading, unless one determines its origin by examining the infant with both sides of the chest free and symmetrical.

In regard to the heart the result of the examination of two hundred and seventy-five cases, it may be concluded that, up to the sixth year, the apex beat is found much more commonly in the fourth intercostal space and in the midclavicular line and that, after this period, it is usually in the fifth space, in or just within the midclavicular line. Occasionally, however, it continues to beat in the fourth space even up to the twelfth year. The average outline of the heart for children under 3 years of age is as follows: upper border, second rib; right border, midsternum; left border, just without the midclavicular line. From the third to the sixth year: upper border, upper border of the third rib; right border, midsternum; left border, in or just without the midclavicular line. From the sixth to the twelfth year: upper border, third rib; right border, from the midsternum to the right edge of the sternum; left border, most commonly in the midclavicular line.

Concerning the venous hum and functional heart murmurs, which few observers mention as occurring in the early years of life, the authors give interesting data. They believe that they bear a close relationship to the general nervous tone of the individual. The venous hum was studied in two hundred and twenty-six cases, twenty-four of which were under 3 years of age. Functional heart murmurs were studied in two hundred and sixty-seven cases, the murmur being best heard in the majority of cases in the pulmonary area. One most significant observation was made: of nineteen children under one year in a children's home, twelve were foundlings and seven breast-fed. Among the foundlings there was found the venous hum four times, a functional murmur seven times. In the seven breast-fed children, there were no venous hums present. One who had recently had a severe attack of measles had a functional murmur.

